# Risk identification and technical modifications reduce the incidence of post-cholecystectomy bile leakage: analysis of 5675 laparoscopic cholecystectomies

**DOI:** 10.1007/s00423-021-02264-z

**Published:** 2021-08-26

**Authors:** Ahmad H. M. Nassar, Hwei Jene Ng

**Affiliations:** 1 Laparoscopic Biliary Surgery Service, University Hospital Monklands, Lanarkshire, Airdrie, Scotland, ML6 0JS UK; 2 NHS Greater Glasgow and Clyde, Glasgow, UK

**Keywords:** Laparoscopic cholecystectomy, Post-cholecystectomy bile leak, Subvesical ducts, Hepatocystic ducts, Cystic duct ligation, Complications, Instruments, Difficulty grading

## Abstract

**Purpose:**

The main sources of post-cholecystectomy bile leakage (PCBL) not involving major duct injuries are the cystic duct and subvesical/hepatocystic ducts. Of the many studies on the diagnosis and management of PCBL, few addressed measures to avoid this serious complication. The aim of this study was to examine the causes and mechanisms leading to PCBL and to evaluate the effects of specific preventative strategies.

**Methods:**

A prospectively maintained database of 5675 consecutive laparoscopic cholecystectomies was analysed. Risk factors for post-cholecystectomy bile leakage were identified and documented and technical modifications and strategies were adopted to prevent this complication. The incidence, causes and management of patients who suffered bile leaks were studied and their preoperative characteristics, operative data and postoperative outcomes were compared with patients where potential risks were identified and PCBL avoided and with the rest of the series.

**Results:**

Twenty-five patients (0.4%) had PCBL (7 expected and less than half requiring reintervention): 11 from cystic ducts (0.2%), 3 from subvesical ducts (0.05%) and 11 from unconfirmed sources (0.2%). The incidence of cystic duct leakage was significantly lower with ties (0.15%) than with clips (0.7%). Fifty-two percent had difficulty grades IV or V, 36% had empyema or acute cholecystitis and 16% had contracted gallbladders. Twelve patients required 17 reinterventions before PCBL resolved; 7 percutaneous drainage, 6 ERCP and 4 relaparoscopy. The median hospital stay was 17 days with no mortality. Hepatocystic ducts were encountered in 72 patients (1.3%) and were secured with loops (54.2%), ties (25%) or sutures (20.8%) with no PCBL. Eighteen sectoral ducts were identified and secured.

**Conclusion:**

Ligation of the cystic duct reduces the incidence of PCBL resulting from dislodged endoclips. Careful blunt dissection in the proper anatomical planes avoiding direct or thermal injury to subvesical and sectoral ducts and a policy of actively searching for hepatocystic ducts during gallbladder separation to identify and secure them can reduce bile leakage from such ducts.

## Introduction

The incidence of post-cholecystectomy bile leakage (PCBL) other than from major ductal injury is 0.3–2.7% of patients undergoing laparoscopic cholecystectomy (LC) [1–3]. The sources of most bile leaks are either the cystic duct stump (Strasberg classification A) or inadvertently injured hepatocystic ducts (HCD) or subvesical ducts (SVD) [3]. This can result in biloma, biliary fistula or in localised or generalised peritonitis. Published studies have addressed the sources of PCBL and their management [1–3] but with less emphasis on measures to prevent this complication.

A variety of laparoscopic instruments and various dissection techniques are used during LC. The traditional laparoscopic technique utilises metal clips for cystic duct occlusion. These come with their unique set of complications, dislodgement and PCBL is but one [4]. Cystic pedicle dissection and separation of the gallbladder from the liver are usually carried out with diathermy hooks. Neither metal clips nor diathermy hooks were used in the open cholecystectomy era.

Unknown numbers of PCBL are subclinical. Others are self-limiting and may not require reintervention. However, some cause significant morbidity and require reinterventions, whether percutaneous (P/C), endoscopic, laparoscopic or by laparotomy [5–8].

The primary aim of this study was to examine the causes and mechanisms of PCBL. The secondary aims were to evaluate the effects of specific preventative strategies, namely a policy of optimising dissection techniques to identify and secure such ducts, avoiding direct and thermal injury, and of occluding the cystic duct using ties rather than metal clips.

## Methods

This biliary firm is dedicated to managing all comers with biliary emergencies during the index admission resulting in a high emergency workload.

Detailed prospective data was collected from all laparoscopic cholecystectomies performed by a single surgeon or his trainees under direct on table supervision over 28 years. Patient demographics, type and cause of admission, American Society of Anaesthesiologists (ASA) classification, operative difficulty grade (Nassar scale) [9, 10], operative time, conversion to open, perioperative complications and 30-day mortality were analysed. Prospective operative documentation of identified hepatocystic, subvesical and extrahepatic sectoral ducts and the strategies used to secure them avoiding PCBL were evaluated and compared to the rest of the patient cohort. Patients who suffered PCBL were identified and data relating to their management was collected and added to the database. Follow-up data of between 2 and 17 years was available. This was carried out by reviewing patients’ computer records on the hospital system. Wound infections may be underreported as some are treated in the community and may not be captured on follow-up documents.

For the purpose of this study, the definition of PCBL was the presence of bile in an abdominal drain or the recovery of bile from the peritoneal cavity by percutaneous drainage or at reoperation, regardless of whether or not this leakage was expected at the time of LC or transcystic bile duct exploration. Post-choledochotomy bile leakage was excluded as it is an occasional occurrence after bile duct exploration through a choledochotomy, whether primary closure or biliary drainage was used, and is usually without consequences due to the utilisation of abdominal drainage. Subvesical ducts (SVD) are those ducts running under the Glisson’s capsule of the cystic plate, usually draining into the anterior right sectoral/sectional duct or the right hepatic duct. Hepatocystic (or hepaticocholecystic) ducts (HCD) are small SVD ducts that drain directly into the gallbladder. Although these ducts have been called ducts of Luschka or accessory ducts in some literature, the current authors have opted to avoid using these terms because they  do not signify any anatomical definition.

This study is not concerned with the origins of SVDs but with the relationship to the gallbladder fossa where dissection in the proper anatomical plane would ensure their preservation under normal circumstances. These are referred to as types 1 and 2 SVD by Schnelldrofer et al. while HCD are classified types 3 and 4. The authors suggested that the term “ducts of Luschka” should be abandoned [11].

Informed consent was obtained from patients with specific emphasis on the specialisation of the unit in single-session management of suspected bile duct stones. Ethical approval was not required as the management protocols were in line with the recommendations of national and international societies.

### Technique

After establishing pneumoperitoneum by open access, a four-port technique is employed in the American position. The cystic pedicle was dissected using a blunt, flat-jawed dissector/grasper, called the “duckbill dissector” (Karl Storz, Tuttlingen, Germany) with occasional use of diathermy to divide adhesions or peritoneum. Once the cystic pedicle anatomy was clarified, initially using the infundibular technique and later displaying the critical view of safety, the gallbladder/cystic duct junction was ligated using 2–0 absorbable suture.

After incising and cannulating the cystic duct, an operative cholangiography was obtained to exclude bile duct stones. The cystic duct was ligated using absorbable 2–0 suture material and divided having abandoned metal clips 23 years ago following a drain-controlled PCBL which resulted from clips not securing a thick and inflamed cystic duct.

**Fig. 1:**
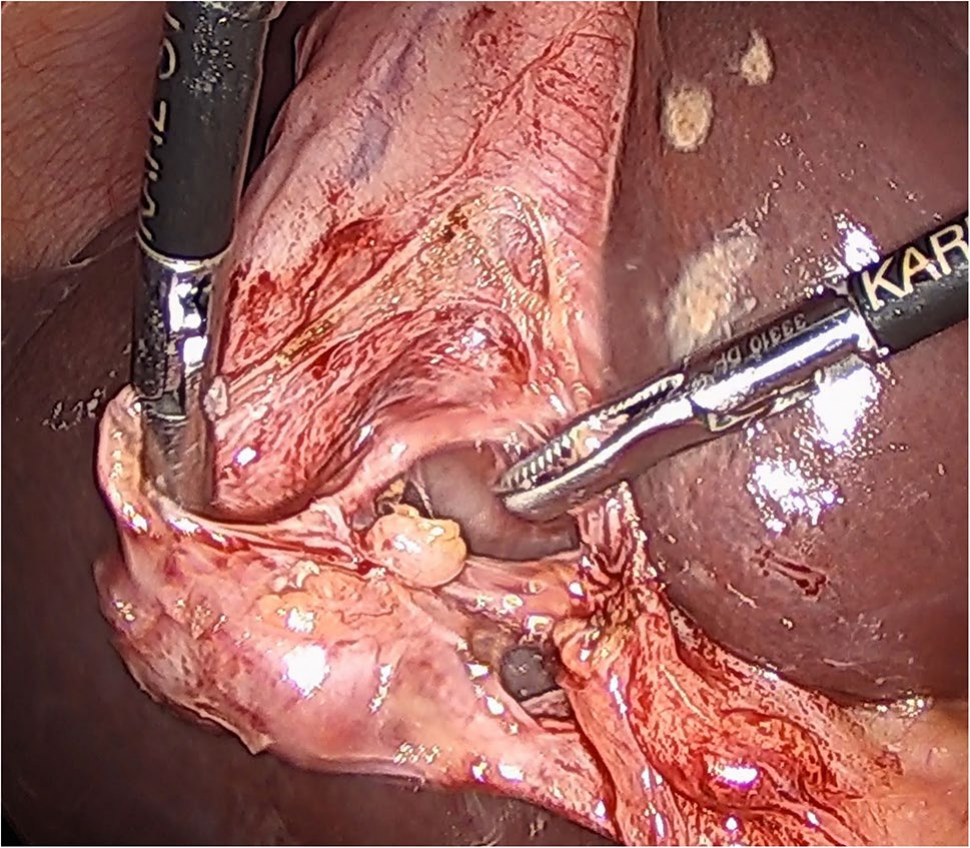
“Duckbill” grasper dissection identifying and isolating a HCD in the gallbladder bed

**Fig. 2:**
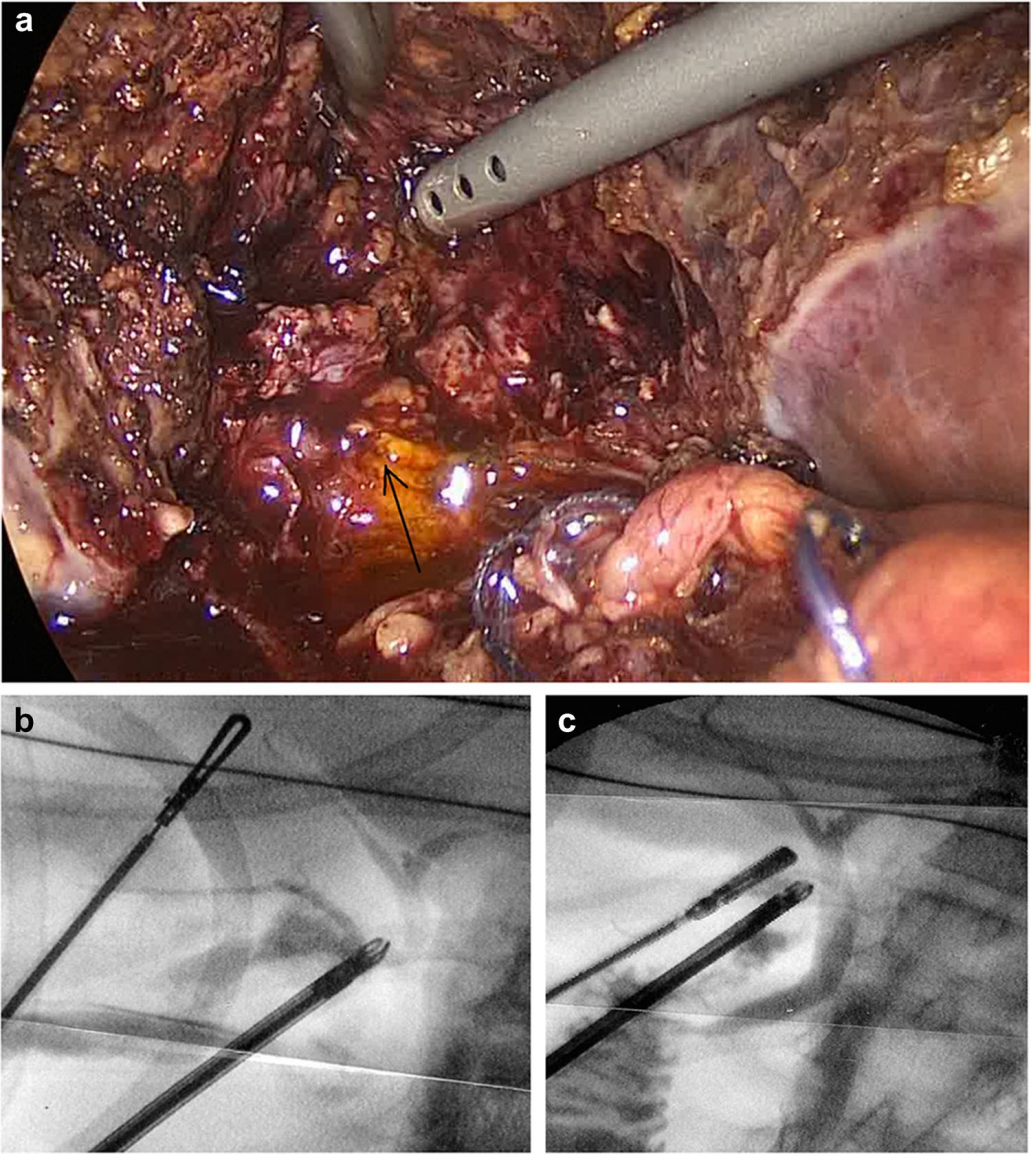
**A** A leaking SVD seen on the gallbladder bed proximally. An empyema of the gallbladder with the thick posterior wall fused with the liver resulted in a breach of the anatomical capsule. **B** The leaking SVD is cannulated and cholangiography obtained. **C** Completion cholangiography confirming no further leakage at the sutured duct

The diathermy hook was not used for dissecting the gallbladder from the liver beyond the first few cases. The “duckbill dissector” was used, opening windows in the peritoneal reflection, the jaws spreading to create a subserosal plane and bluntly sweeping the gallbladder wall away from the liver. This makes it possible to identify any tubular structures between the liver and the gallbladder without using diathermy (Fig. 1). Any ducts found running from the cystic plate to the gallbladder (HCDs) or producing bile in the gallbladder bed (SVDs) are ligated, looped or sutured using absorbable 2–0 suture material. Careful inspection of the gallbladder bed for any sign of leaking bile is carried out during and after separation of the gallbladder, particularly where an acutely inflamed or a contracted gallbladder is encountered, with the loss of a distinctive plane necessitating the use of scissors or the spatula function of the duckbill grasper, and at a second inspection following the closure of the umbilical port site. Any bile seen during dissection is investigated to exclude or confirm a leak and any leaking point (Fig. 2A) is cannulated where possible and cholangiography obtained to confirm the distribution of the leaking subvesical or hepatocystic duct (Fig. 2B). The leaking point is then secured using a loop ligature or sutures and a completion cystic duct cholangiography is performed to demonstrate an intact biliary tree (Fig. 2C). Drains were only used when SVD/HCD needed to be secured with sutures.

### Statistical analysis

Categorical variables are expressed as *n* and percentage (%) and continuous as median. For comparison between PCBL and no PCBLgroups, differences were assessed with Student T test for continuous variables and chi-square or Fisher exact test for categorical variables. P value of < 0.05 was considered statistically significant. All analyses were performed using IBM SPSS 22.

## Results

Five thousand six hundred seventy-five patients underwent laparoscopic cholecystectomy performed by a single surgeon or by trainees under on-table supervision between February 1992 and December 2019. The male:female ratio was 1:2.9 with a median age of 51 years (8 to 91 years). 44.3% were emergency admissions.

PCBL was documented in 25 patients (0.4%). These had a significantly higher incidence of emergency admissions, acute cholecystitis/empyema found at operation and a higher operative difficulty grade than the rest of the series (Table 1). Although routine display of CVS was only adopted in 2016 as part of a prospective study of the causes of failure to achieve the CVS [12], a review of the operative records of earlier PCBL patients showed that displaying the CVS failed in 14 of 25 LCs (56%) (Table 2). Thirteen had difficulty grades IV or V, 9 had empyema or acute cholecystitis, 4 had contracted gallbladders and 4 of the 14 had wide cystic ducts with impacted stones. Five required suturing of the CD stump.

**Table 1:** Patient demographics and perioperative data of the whole series and post-cholecystectomy bile leak (PCBL)

Characteristics	No PCBL (n = 5650) (%)	PCBL (n = 25) (0.4%)	p value
Age, median (range)	51 (8–91) years	62 (34–89 years)	
Gender			p = 0.034
Male	1450 (25.7%)	12 (48.0%)	
Female	4189 (74.1%)	13 (52.0%)	
No record	11 (0.2%)	-	
Type of admission			p < 0.001
Elective	3151 (55.8%)	10 (40%)	
Emergency	2493 (44.1%)	15 (60%)	
Not recorded	6 (0.1%)	-	
Condition of gallbladder			p = 0.001
Chronic inflammation	3884 (68.7%)	10 (40.0%)	
Acute cholecystitis	343 (6.1%)	3 (12.0%)	
Empyema	393 (7.0%)	8 (32.0%)	
Contracted	696 (12.3%)	4 (16.0%)	
Mucocele	334 (5.9%)	-	
Operative difficulty grade			p < 0.001
Grade I	1874 (33.2%)	6 (24.0%)	
Grade II	1724 (30.5%)	3 (12.0%)	
Grade III	1138 (20.1%)	1 (4.0%)	
Grade IV	799 (14.1%)	14 (56.0%)	
Grade V	110 (2.0%)	1 (4.0%)	
No record	5 (0.1%)	-	
Intraoperative cholangiography	5196 (92.0%)	22 (88.0%)	p = 0.768
Abdominal drains	2886 (51.1%)	19 (76%)	p = 0.001
Duration of operation, median (range)	60 (15–570) min	95 (35–285) min	
Open conversion	28 (0.5%)	0	
Duration of hospital stay, median (range)	4 (1–160) days	17 (6–49) days	
Perioperative complication rate	191 (3.4%)	3 (12.0%)	p = 0.061
30-day readmission rate	151 (2.7%)	8 (32.0%)	p < 0.001

**Table 2:** PCBL after failed CVS. *GB* gallbladder, *CD* cystic duct, *HP* Hartmann pouch, *CBD* common bile duct, *TCE* transcystic exploration

Case no	Age	GB and pedicle condition	Difficulty grade	LC/TCE	CD closure	Drain	Source of PCBL	Hospital stay/days	Management
1	55	Contracted, wide CD with stone	IV	TCE	Sutured	Yes	CD stump	6	Settled conservatively
2	49	Contracted, wide CD with stone	III	TCE	Clips	Yes	CD stump	9	ERCP and stent
3	89	Acute cholecystitis, wide CD	IV	LC	Clips	Yes	Unknown	?	Settled conservatively
4	70	Wide CD with stone, longitudinal incision	IV	LC	Sutured	Yes	CD stump	6	Settled conservatively
5	75	Contracted GB, thick pedicle, artery fused with duct	IV	TCE	Sutured	Yes	CD stump	9	Settled conservatively
6	62	Contracted GB, cirrhosis, dilated veins at pedicle	IV	TCE	Ties	Yes	?biliary drain/CD	?	P/C drainage
7	78	Cholecystoduodenal fistula, acute	IV	LC	Ties	Yes	Unknown	12	ERCP and stent
8	56	Empyema	IV	LC	Sutured	Yes	CD stump	30	Settled conservatively
9	77	Empyema	IV	TCE	Ties	Yes	CD stump	34	Settled conservatively
10	53	Empyema	IV	LC	Ties	Yes	CD stump	26	P/C drainage
11	68	Empyema	IV	TCE	Ties	Yes	?biliary drain/CD	64	ERCP, P/C drainage
12	72	Empyema, abscess Liver, HP to CBD	IV	TCE	Ties	Yes	Unknown	49	P/C drainage
13	80	Empyema, GB abscess into liver	V	LC	Ties	Yes	?biliary drain/CD	22	Settled conservatively
14	75	Empyema, wide CD with stone, friable	IV	TCE	Sutures	Yes	CD stump	21	Settled conservatively

Three quarters of all patients suffering PCBL had had abdominal drains inserted at the time of LC and bile presented through the drains. Abdominal drains were inserted in 51% of patients who had no PCBL. This low threshold for drainage was due to the high incidence of emergency procedures (44%) and of bile duct explorations (23%) resulting from the unit’s interest in index admission surgery for biliary emergencies. No open conversions resulted from intraoperative bile leakage. Two cases of bile duct injuries occurred in the whole series but were not converted. They were recognised, stents and drains were inserted, and the patients referred to a liver surgery unit for reconstructive surgery within 24 h. The causes of conversion in the whole series have previously been published [13].

**Fig. 3:**
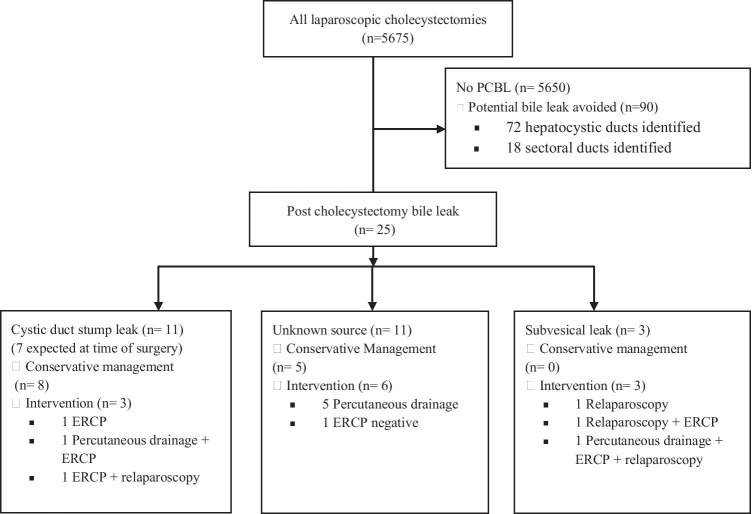
Flow diagram illustrating sources and management of post-cholecystectomy bile leaks and patients where potential risk of leakage was avoided

The source of bile leakage was likely to be the cystic duct stump in 11 patients, although reintervention was needed in only 3. Seven patients had expected CD stump leakage: six due to friable, short or wide cystic duct stumps sutured at the time of LC and one due to a perforated CD stump during failed cholangiography. All had drains inserted at the time of surgery which contained the leakage and remained asymptomatic until the leakage resolved within a median of 4 days (range 3–10). One patient needed ERCP (last patient where metal clips were used), one required USS-guided percutaneous drainage and ERCP and one an ERCP followed by relaparoscopy (Fig. 3). The incidence of PCBL dropped from 0.7% (3/413) during the early part of the study when metal clips were used to 0.15% (8/5262) after adopting tie ligatures (p = 0.011).

### Reintervention

Thirteen patients suffering PCBL settled without requiring any form of imaging or treatment and the sources of leakage were never confirmed. Twelve patients required 17 reinterventions, eight needing only one intervention. Four of the 12 requiring reinterventions were still in hospital when PCBL presented. Eight were readmitted 2 to 10 days postoperatively with abdominal pains and only one with early peritonitis requiring relaparoscopy on the same day. Four patients had bile leakage continuing after one intervention necessitating further treatment. The sources of PCBL in the four patients requiring relaparoscopy are shown in Table 3.

**Table 3:** PCBL patients requiring reinterventions: timing of discharge and readmissions. *LC* laparoscopic cholecystectomy, *I/P* inpatient, *IOC* intraoperative cholangiogram, *N/A* not available

LC	Difficulty grade	Discharge postop day	Readmission postop day	Presentation	P/C day	ERCP day	Relaparoscopy	Cause and management	Hospital stay
Patient 1	III	I/P	I/P	PCBL in drain	-	2	-	CD leak, stent	6
Patient 2	IV	I/P	I/P	PCBL in drain	10	-	-	Subcapsular collection. Settled	N/A
Patient 3	IV	I/P	I/P	PCBL in drain	-	6	-	ERCP negative. PCBL stopped	12
Patient 4	II	1	2	Peritonitis	-	-	2	SVD. IOC, suture	7
Patient 5	I	1	8	Abdominal pain	21	-	-	Settled, unknown source	18
Patient 6	I	1	4	Abdominal pain	6	7	8	SVD, IOC, suture	20
Patient 7	IV	I/P	I/P	PCBL in drain	14	-	-	Settled, unknown source	26
Patient 8	IV	28	32	Abdominal pain	18	22	-	CD stump. Settled after further P/C drain on day 45	64
Patient 9	I	1	6	Abdominal pain	14	-	-	Settled, unknown source	36
Patient 10	IV	1	10	Abdominal pain	18	-	-	Settled, unknown source	49
Patient 11	I	2	4	Abdominal pain	-	8	4	SVD no IOC, postop ERCP	16
Patient 12	I	1	4	Abdominal pain	-	14	15	CD leak, no IOC	31

The median overall postoperative stay for PCBL patients was 17 days, compared to 4 days for the whole series. The morbidity rate, incidence of readmissions and reinterventions were all significantly higher in the PCBL cohort (Table 1). There was no mortality in this group. Following initial follow-up, a review of the hospital computer records showed no biliary problems of between 2 and 17 years (a median follow-up of 5 years).

Ninety patients (1.6%) with hepatocystic and sectoral ducts at risk of potential PCBL were identified at surgery and preventative technical measures implemented, avoiding direct or thermal injury and subsequent bile leaks. Of these, hepatocystic ducts were encountered during gallbladder dissection in 72 patients and confirmed to enter the gallbladder wall. All were carefully dissected away from the gallbladder, encircled and found to produce bile upon incision. Thirty-nine (54.2%) were secured with endoloops, 25% with intracorporeal ties and 20.8% were sutured. Any additional tubular structures not producing blood when incised where also ligated but were not recorded as HCDs. No PCBL occurred from any of the ducts identified and secured during LC. Preoperative ERCP had been done in four patients and operative cholangiography was carried out in 66 of the 72 patients; none positively identifying these ducts. The median length of surgery was 70 min. The hospital stay, morbidity and readmission rate in this cohort were comparable to the rest of the series.

Eighteen extrahepatic sectoral ducts were encountered during dissection of the cystic pedicle. These were clearly visible near the cystic duct junction with the common hepatic duct [14, 15], were confirmed on transcystic duct cholangiography and avoided during the separation of the gallbladder. Two were found to enter the neck of the gallbladder/ cystic duct (type F Couinaud classification) and needed to be ligated once cholangiography confirmed the integrity of the main bile ducts. No consequences were reported on follow-up for 3 and 4 years.

A further twenty patients had drain-controlled bile leakage following bile duct explorations through a choledochotomy. Biliary drainage was established with T-tubes in 8 patients and transcystic tubes in 7 and primary closure of the choledochotomy was carried out in 5. However, these did not meet the definition of PCBL in the literature and were therefore excluded from further analysis.

No PCBL in this study resulted from main bile duct injury. Two ductal injuries were diagnosed at the time of operation. In one, a short cystic duct entered the right hepatic duct, which was opened to perform a cholangiography but found to join the left hepatic duct close to the duodenum to form the common bile duct. In the other, a thin common hepatic duct was mistaken for a small vessel crossing between the cystic artery and the cystic duct and cauterised. The injuries were detected and confirmed on cholangiography before the ducts were stented laparoscopically and the patients referred for reconstructive surgery within 24 h.

## Discussion

Although some complications became more frequent in the laparoscopic era than with open cholecystectomy (vascular, intestinal and biliary injuries) [16–18], improved technology and refined skills reduced their incidence. However, PCBL still occurs in 0.3–2.7% of patients [1–3] compared to 0.5% with open cholecystectomy [19], significantly increasing the morbidity and healthcare costs of LC [20].

PCBL can result from injury to SVD or division of HCD during gallbladder separation and may not be evident at the time of LC where the diathermy hook is used, dividing and coagulating an invisible duct with no immediate bile leakage. This mechanism does not allow the surgeon to identify a tubular structure or determine whether it is a duct or a blood vessel, making it difficult to remedy a bile leak. The stoma of a coagulated duct can later lead to PCBL which may be subclinical or present in various ways. Biloma formation, biliary peritonitis, biliary fistula or generalised sepsis may result in significant morbidity if not identified and treated appropriately in a timely fashion [21]. Cystic stump leaks can occur from faulty clip application, slipping of the clips or direct injury to the cystic duct stump during dissection in 0.12–0.26% [22]. As hook diathermy was not used throughout this study, no comparative analysis would be possible. However, one of three consequential CD leaks resulted from metal clips applied to a thick inflamed cystic duct early in the series. The leak was controlled due to the use of an abdominal drain and ERCP was carried out when the leak persisted. The recognition of these aetiological mechanisms has not resulted in changing current practice and the incidence of PCBL does not seem to decline. Further randomised studies are required to study the relationship between different techniques of securing the cystic duct and of gallbladder dissection and the occurrence of PCBL.

Bile leakage classically presents within a few days of LC but can be diagnosed up to 30 days later. Symptoms can be nonspecific (abdominal pain, fever, nausea and/or vomiting) and be attributed to other postoperative complications [23, 24].

Only one-half of our PCBL cohort required US, CT or MRI scans showing fluid collections. These may be subhepatic, intrahepatic, subcapsular or rarely in the retroperitoneal space [25, 26]. HIDA scans may show continuity between a biloma and the biliary tree [25–29]. However, ERCP and percutaneous transhepatic cholangiography (PTC) are the most accurate at demonstrating the exact site of a leak [5–7].

Several treatment options were used to drain collections and prevent further leakage including initial image-guided percutaneous drainage (the only intervention necessary in 40% of our reinterventions), ERCP with sphincterotomy and/or biliary stenting (alone in 16%) or a combination of these techniques [5–8]. Ahmad et al. [28] recommended an algorithmic approach using percutaneous drainage followed by endoscopic stent placement for persistent leakage or sepsis and initial endoscopic treatment only if no significant abdominal collection is present.

**Fig. 4:**
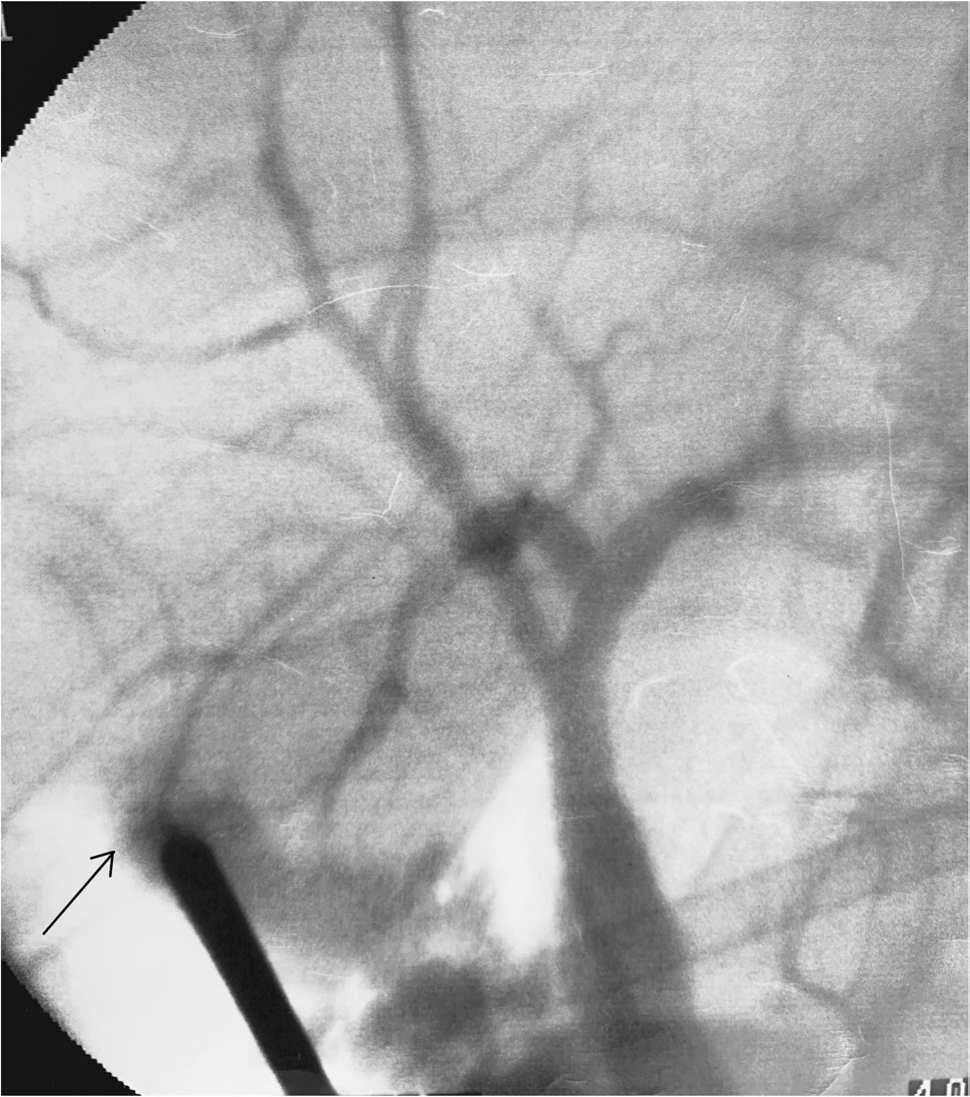
A leaking SVD seen at relaparoscopic exploration is cannulated (tip of cannula at opening) and a cholangiography obtained to confirm its distribution and the integrity of the main ducts

Relaparoscopy, peritoneal lavage and drain placement with or without cholangiography and securing the source of leakage were necessary in four patients, an approach advocated for patients with inadequate drainage, localised or generalised peritonitis and biliary sepsis [30]. Once a cystic duct leak is excluded, a thorough search of the gallbladder bed can identify a leaking SVD which may be cannulated to obtain an operative cholangiography (Fig. 4). This will aid effective closure of the leaking duct, minimising the duration of postoperative drainage required. Cholangiography confirms the integrity of the main bile ducts avoiding further endoscopic interventions.

In spite of the many studies describing treatment options, few have addressed measures to prevent PCBL. Wills et al. used the Harmonic® scalpel (Ethicon Endo-Surgery, Cincinnati, OH) for cystic duct occlusion but their PCBL rate proved to be higher than that with clips [31]. Locking absorbable clips have a reduced cystic duct leak rate compared with metal clips, are not associated with artefacts on subsequent imaging and have a reduced incidence of migration [32]. Sutures, three-throw reef knots and Roeder slip knots [32, 33] have also been used. However, a Cochrane review [33] on best available cystic duct occlusion did not conclude in favour of any method due to inadequate samples.

The low rates of PCBL with open cholecystectomy [19] may be due to blunt dissection of the cystic pedicle, traditional ligation of the cystic duct and gall bladder separation with little or no diathermy. In our practice, cystic duct ligation with absorbable ties in the last 5250 LC over 23 years reduced the incidence of PCBL confirmed to be from the cystic duct stump to less than 0.2%. A large single institution series also suggested that intracorporeal ligation considerably reduced cystic duct leakage [34].

Although some authors warned that laparoscopic knots were weaker than the open hand tied knots [35], Lopez et al. disproved this in a double-blinded study comparing the two methods [36]. The first author has previously recommended intracorporeal ligation and suturing as essential skills for advanced laparoscopic surgical procedures [37] and demonstrated the feasibility of intracorporeal ligation of the cystic artery and duct in the initial phase of training within 3.5 min [38]**.**

**Fig. 5:**
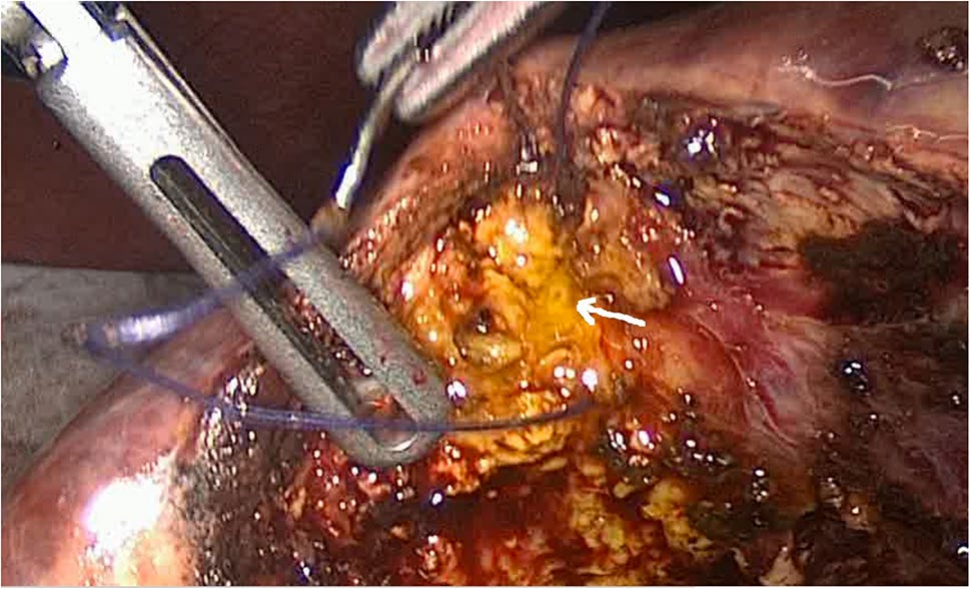
A leaking SVD on the distal gallbladder bed is sutured

Subvesical ducts are present in 3–5% of autopsies [39, 40]. They originate from the right hepatic lobe, course along the gallbladder fossa and usually drain into extra hepatic bile ducts. They may also drain into the gallbladder in over 40% of cases [11]. They can occur anywhere from very close to the cystic duct to the fundus of the gallbladder (Fig. 5). As injury to SVDs is the second most frequent cause (15–20%) of PCBL [41], it has been suggested that their preoperative diagnosis was possible using drip-infusion cholangiography with computed tomography (DIC-CT) [42, 43]. However, such techniques are not of practical value. In this series, SVD/HCD were unlikely to be identified on preoperative imaging or even intraoperative cholangiography. Of 72 patients, four had had preoperative ERCP and 3 had MRCP, none positively identifying such ducts on retrospective review. It is therefore important to be aware of their potential presence during the operation and to keep gallbladder dissection in the proper anatomic plane. The Glisson capsule may be breached during dissection when an empyema or contracted gallbladder distorts that plane (Figs. 2A and 5). Various salvage strategies can be used to overcome such difficulties. In the whole series, fundus first dissection was used in 173 patients (3%) with PCBL occurring in only two, with relaparoscopy and ERCP required in one patient each and one settling conservatively. Six patients (0.1%) underwent subtotal cholecystectomy, PCBL occurring in only one where the fenestrating type was employed. The gallbladder bed should be carefully inspected, and should a leak be suspected or confirmed, the leaking point should be secured. The use of diathermy should be minimised as it may mask any bile leakage during surgery making PCBL more likely.

**Fig. 6:**
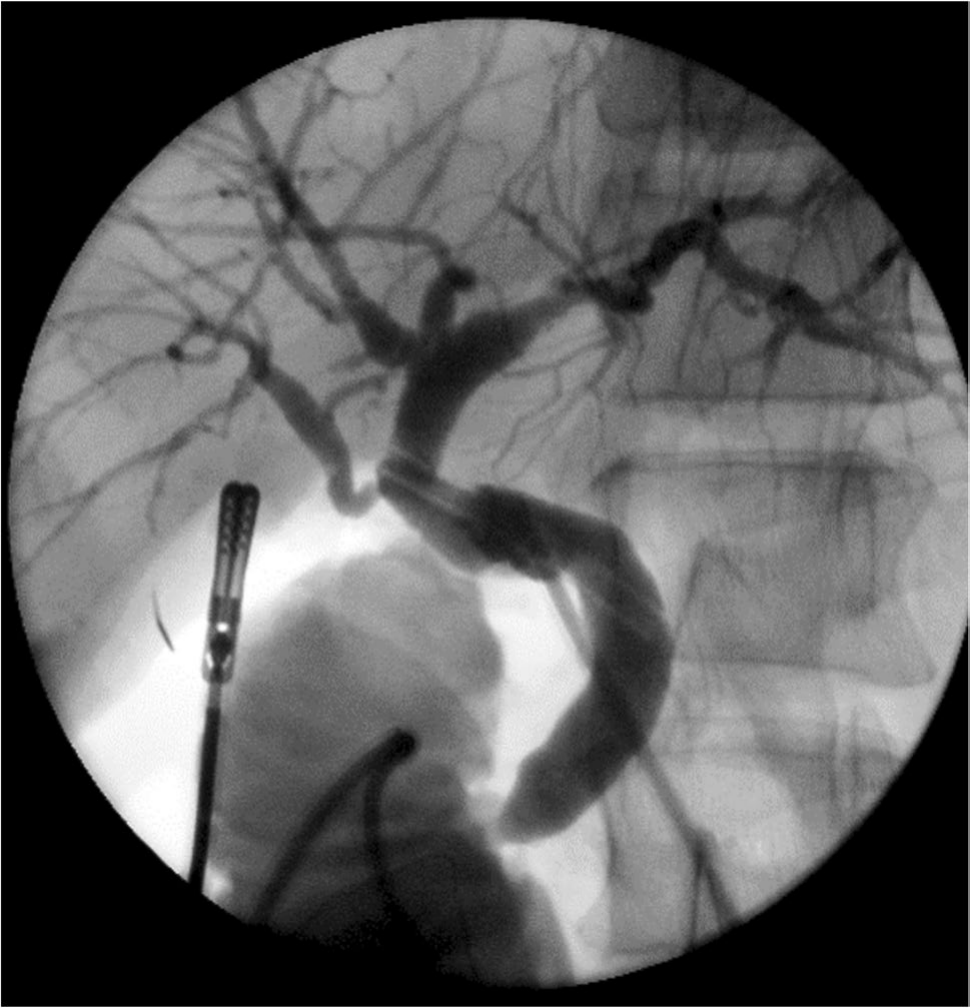
Cystic duct cannulation and cholangiography showing a large right posterior sectional duct joining the common hepatic duct. Obscured by the gallbladder, this was extrahepatic and can be at risk of injury during gallbladder dissection

Sectoral ducts are more likely to be identified on MRCP [44, 45]. However, preoperative MRCP is not routine practice in our unit as we use single-session laparoscopic management of bile duct stones. Two of 18 sectoral ducts detected at operation had had preoperative MRCP with easily identifiable ducts. However, during surgery, all 18 ducts were clearly visible in the CVS and on cholangiography. Cholangiography identification may precede visual identification of significant sectoral ducts not readily visible during initial dissection even after achieving the CVS (Fig. 6). It is important, therefore, for dissection to proceed as close to the gallbladder wall as possible for a safe distance from the cystic pedicle in order to ensure the preservation of such ducts. The correct choice of dissection instruments, whether diathermy is utilised and the experience and skill of the surgeon, will determine the outcome of the dissection.

Although diathermy hook dissection is one of the main differences between open and laparoscopic cholecystectomy, its possible role in PCBL has never been addressed. A European Association for Endoscopic Surgery (EAES) survey conducted in 2003 concluded that responders did not believe a link existed between the dissection instrument and PCBL. However, most surgeons suggested that securing the cystic duct with ties was associated with less bile leakage [46].

A study of the identification and categorisation of technical errors conducted by Tang et al. [47] with Observational Clinical Human Reliability Assessment (OCHRA) in 200 LC showed more errors and a higher error probability using the electrosurgical hook compared to dissection forceps. Sixty-eight percent of errors resulted from failure to visualise the tip of the hook and more “consequential” errors and serious injuries were associated with it. Applying excessive force and wrong instrument direction/spatial orientation resulted in 53% and 42% of the errors committed using the hook. Tang et al. concluded that “the poor design of the electrosurgical hook knife is largely responsible for the error modes.” Our high rate of HCD detection and preservation (1.26%) and very low rate of PCBL confirmed to be from SVD (0.05%) may be the result of not using diathermy hook dissection. Randomised studies are required to compare the safety of instruments used during LC and to identify risk factors associated with them.

The incidence of PCBL in this study was 0.4% with less than half requiring any reintervention. Specific risk factors identified include acutely inflamed or contracted gallbladders causing their dissection to breach Glisson capsule and large stones impacted in wide cystic ducts which may necessitate longitudinal incision to remove them and the cystic duct stump to be sutured. The study highlights the importance of abdominal drainage in at-risk cases, reducing the incidence of serious complications and the need for reintervention, with most settling spontaneously.Less than half required reinterventions and less than a third needed ERCP or relaparoscopy. A comparison between our results and some published studies is shown in Table 4. The total rates of PCBL as well as the rate of leakage from the cystic stump and SVD were all significantly lower than other series (p < 0.001).

**Table 4:** Comparison of PCBL rates, sources and management in some published studies

Studies	PCBL rate (exclude major bile duct injury)	Source			Management	Mortality
		Cystic duct	Subvesical duct	Unknown source		
Current study (N = 5675)	25 (0.4%)	11 (0.2%)	3 (0.05%)	11 (0.2%)	ERCP, percutaneous drainage, relaparoscopy	0
Merrie et al. [2] (N = 929)	18 (1.9%)	N/A	N/A	N/A	ERCP, percutaneous drainage, reoperation	1
Kozarek et al. [5] (N = 597)	3 (0.5%)	3 (0.5%)	N/A	N/A	ERCP, surgical intervention	N/A
Sinha et al. [22] (n = 756)	5 (0.7%)	4 (0.5%)	N/A	1	ERCP	N/A
Ahmad et al. [30] (n = not documented)	24	10	2	12	ERCP, percutaneous drainage, surgical intervention	1
Shaikh et al. [32] (n = 2011)	13 (0.6%)	9 (0.4%)	3 (0.15%)	1 (0.05%)	ERCP, percutaneous transhepatic cholangiography	N/A
Goswami et al. [40] (n = 1190)	14 (1.2%)	1 (0.1%)	3 (0.25%)	10 (0.8%)	Operative intervention	2

### Prevention strategies

Our CD leaks declined significantly after abandoning closure with metal clips and adopting a policy of suture ligation of the cystic duct. This is consistent with a systemic review of cystic duct closure techniques by van Dijk et al. concluding that cystic stump leakage was 0% for ligatures and locking clips and 1% for metallic non-locking clips [48]. This 28-year study is unique in having prospectively adopted and documented a policy of actively searching for and securing hepatocystic ducts without diathermy, adhering to an anatomical plane close to the gallbladder wall to avoid SVD injuries in every LC and identifying and protecting sectoral ducts through being aware of their anatomical configurations, careful analysis of available preoperative imaging, and performing cholangiography.

### Limitations

This series is based on the practice of a single surgeon who had a few years’ experience in open cholecystectomy prior to the introduction of laparoscopic surgery, allowing awareness of the entity of PCBL, although its incidence resulting from SVDs and HCDs was not well established. A special clinical interest in managing biliary emergencies and a research interest in the prevention of PCBL through modified dissection and ligation techniques may not be representative of average surgical practice.

## Conclusions

The introduction of unconventional instruments may be responsible for the increased incidence of PCBL in the laparoscopic era compared to open cholecystectomy. Male sex, emergency admission, acute cholecystitis and a higher operative difficulty grade are significant risk factors. The elimination of misapplied or dislodged cystic duct clips reduced the incidence of PCBL from 0.7% during the early part of this study when metal clips were used to 0.15% after adopting suture ligatures. A careful search for hepatocystic ducts during gallbladder separation and securing them without diathermy avoided potential thermal injury to 72 ducts. 0.2% had PCBL needing any reintervention (only 0.07% by relaparoscopy). Although it may be impossible to completely eliminate PCBL, understanding the causative mechanisms and the adoption of preventive measures can minimise patient morbidity and the healthcare costs associated with it.
